# Systemic Sclerosis (SSc) After COVID-19: A Case Report

**DOI:** 10.7759/cureus.23179

**Published:** 2022-03-15

**Authors:** Arjun Chandra, Bashar Kahaleh

**Affiliations:** 1 Internal Medicine, University of Toledo, Toledo, USA; 2 Rheumatology, University of Toledo, Toledo, USA

**Keywords:** netosis, nets, autoimmunity, molecular mimicry, covid-19, sars-cov-2, systemic sclerosis

## Abstract

Since the start of the global pandemic caused by coronavirus disease 2019 (COVID-19), there have been numerous reports of autoimmune and rheumatological disorders developing after infection with SARS-CoV-2. To date, there has been only one reported case of systemic sclerosis (SSc) developing after SARS-CoV-2 infection. Here, we present another case of SSc developing after infection with SARS-CoV-2.

A 48-year-old female with past medical history of anxiety and depression presented to the rheumatology clinic after being referred for further evaluation of abnormal labs, Raynaud’s phenomenon, and other concerning symptoms. Shortly after hospitalization for COVID-19 pneumonia, she began experiencing symptoms that included fatigue, xerostomia, dysphagia, bilateral lower extremity weakness, dyspnea with exertion, unintentional weight loss, and diffuse skin hyperpigmentation. Labs ordered shortly before presentation were significant for antinuclear antibody (ANA) titer > 1:1280. Physical exam was remarkable for puffy fingers, sclerodactyly of the fingers, diffuse skin hyperpigmentation, and abnormal nailfold capillaries. Anti-RNA polymerase III, anti-Scl-70, anti-centromere, anti-SSA, anti-SSB, anti-Smith, and anti-Smith/RNP antibodies were all negative. BNP, aldolase, and serum myoglobin levels were within normal limits while creatine phosphokinase level was slightly decreased. Pulmonary function testing showed reduced diffusion capacity with normal lung mechanics and volumes. High-resolution CT scan of the chest showed interstitial lung disease, with findings suggestive of nonspecific interstitial pneumonia. Transthoracic echocardiogram showed mild elevation of right ventricular systolic pressure, but pulmonary hypertension was not found on right heart catheterization. Esophagogastroduodenoscopy (EGD) with biopsy performed for evaluation of esophageal dysphagia showed sliding hiatal hernia, irregular Z-line, and gastric hyperemia. Biopsy of the distal esophagus was consistent with Barrett’s esophagus. The patient was diagnosed with SSc according to the 2013 American College of Rheumatology/European League Against Rheumatism (ACR-EULAR) classification criteria for SSc. She is currently being treated with mycophenolate mofetil, amlodipine, methotrexate, and prednisone.

## Introduction

Systemic sclerosis (SSc) is an autoimmune disease characterized by vasculopathy and fibrosis of the skin and internal organs. Although it is uncommon, SSc has significant mortality and morbidity [1]. According to the 2013 American College of Rheumatology/European League Against Rheumatism (ACR-EULAR) classification criteria for SSc, skin thickening of the fingers of both hands extending proximal to the metacarpophalangeal joints is sufficient to classify patients as having SSc. If this is not present, patients are considered to definitively have SSc if they score a total of 9 or more based on the following seven additive items, each of which have varying weights: skin thickening of the fingers, fingertip lesions, telangiectasia, abnormal nailfold capillaries, pulmonary arterial hypertension and/or interstitial lung disease, Raynaud’s phenomenon, and SSc-related autoantibodies [2]. Autoantibodies in SSc include anti-centromere, anti-Scl-70, and anti-RNA polymerase III antibodies [1].

It is widely thought that SSc develops in individuals with a permissive genetic background. Evidence that an interferon signature exists in most patients with SSc suggests that viral infections may trigger the development of SSc in genetically predisposed individuals; in fact, development of SSc shortly after acute infection with human cytomegalovirus has been reported [3,4]. Since the start of the global pandemic caused by coronavirus disease 2019 (COVID-19), there have been numerous reports of autoimmune and rheumatologic disorders developing after infection with SARS-CoV-2. Examples include systemic lupus erythematous (SLE), Henoch-Schoenlein purpura, multiple sclerosis, and Guillain-Barré syndrome [5]. It is thought that SARS-CoV-2 infection can trigger the development of autoimmune disease through mechanisms like molecular mimicry, bystander killing, epitope spreading, viral persistence, and formation of neutrophil extracellular traps that lead to exposure of autoantigens [6]. To date, there has been only one reported case of SSc developing after SARS-CoV-2 infection [7]. Here, we present another case of SSc developing after infection with SARS-CoV-2.

## Case presentation

**Figure 1:**
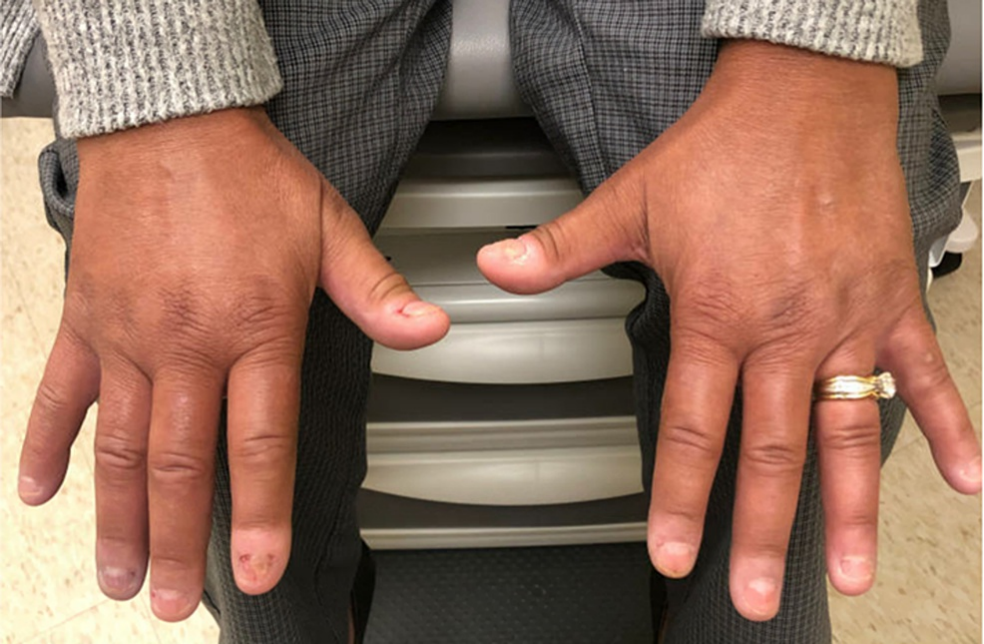
Physical exam findings of puffy fingers, sclerodactyly of the fingers, and skin hyperpigmentation

**Figure 2:**
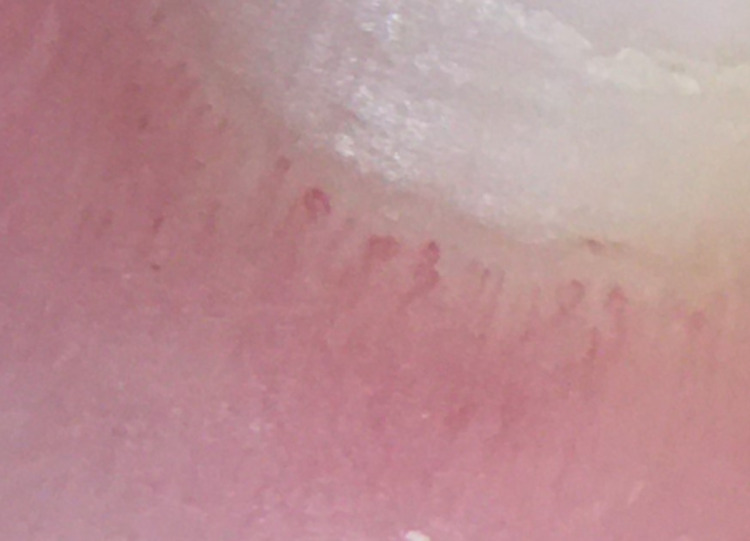
Nailfold capillaroscopy showing a classic systemic sclerosis (SSc) capillary pattern with frequent giant capillaries, mild disorganization of the capillary architecture, and moderate loss of capillaries

**Figure 3:**
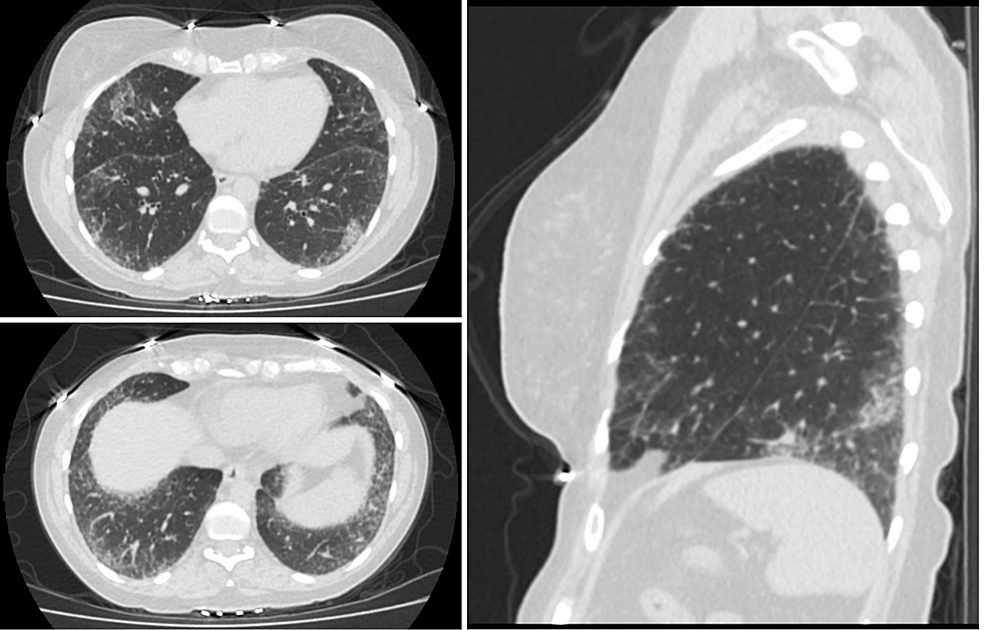
High-resolution CT scan of the chest—axial (top and bottom left) and sagittal (right) views showing lower lobe peripherally predominant lung fibrosis with septal thickening and foci of bronchiectasis, scattered areas of ground glass opacification superimposed within the fibrotic changes, and absence of honeycombing.

A 48-year-old female with past medical history of anxiety and depression initially presented to the rheumatology clinic in August 2021 after being referred by Vascular Medicine for further evaluation of abnormal labs, Raynaud’s phenomenon, and other concerning symptoms. She had been in good health until December 2020 when she was hospitalized for several days for acute hypoxic respiratory failure secondary to COVID-19 pneumonia. Shortly afterwards, she started to experience the following symptoms: persistent fatigue, xerostomia, dysphagia, bilateral lower extremity weakness, Raynaud’s phenomenon, joint pain in both hands that improved with activity and worsened with rest, morning stiffness lasting longer than one hour, dyspnea with exertion, diarrhea, painless oral ulcers, unintentional weight loss of approximately 30 pounds since December 2020, and diffuse skin hyperpigmentation. She denied past history of miscarriages, fevers, chest pain, dry eyes, or alopecia. Labs ordered shortly before presentation were significant for antinuclear antibody (ANA) titer > 1:1280. Physical exam was remarkable for puffy fingers, sclerodactyly of the fingers, diffuse skin hyperpigmentation, and abnormal nailfold capillaries (Figures 1, 2). Anti-RNA polymerase III, anti-Scl-70, anti-centromere, anti-SSA, anti-SSB, anti-Smith, and anti-Smith/RNP antibodies were all negative. BNP, aldolase, and serum myoglobin levels were found to be within normal limits while creatine phosphokinase level was slightly decreased at 29 IU/L. Pulmonary function testing performed in June 2021 showed reduced diffusion capacity of 44% predicted with normal lung mechanics and volumes. High-resolution CT scan of the chest showed interstitial lung disease, with findings suggestive of nonspecific interstitial pneumonia (Figure 3). Transthoracic echocardiogram showed left ventricular ejection fraction of 60% and mildly elevated right-sided pressures with estimated right ventricular systolic pressure of 36 mmHg. However, right heart catheterization showed mean pulmonary artery pressure of 20 mmHg with normal wedge pressure and pulmonary vascular resistance (PVR) of 1.9 wood units. Esophagogastroduodenoscopy (EGD) with biopsy was performed for evaluation of esophageal dysphagia and showed the following: sliding hiatal hernia, irregular Z-line, and gastric hyperemia. Biopsy of the distal esophagus showed squamocolumnar mucosa with intestinal metaplasia consistent with Barrett’s esophagus, and biopsy of the proximal esophagus showed benign squamous epithelium with no evidence of eosinophilic esophagitis.

Since her initial evaluation in August 2021, the patient has followed-up in the rheumatology clinic four times, most recently in January 2022. She is currently taking mycophenolate mofetil 1,500 mg twice daily for treatment of interstitial lung disease. She is also taking amlodipine 5 mg daily for treatment of Raynaud’s phenomenon, as well as methotrexate 12.5 mg once weekly and prednisone 5 mg twice daily for treatment of inflammatory polyarthritis likely related to SSc. She has experienced improvement of her joint pain since starting methotrexate and prednisone and also reports improvement of Raynaud’s phenomenon since starting amlodipine. However, she reports that she has recently been experiencing dry cough and worsening dyspnea with minimal activity. Lung perfusion scan was performed and was normal. She is planning to undergo further evaluation with repeat pulmonary function testing.

## Discussion

According to the 2013 ACR-EULAR classification criteria for SSc, our patient is considered to definitively have SSc since she scored a total of 11 based on the following: sclerodactyly of the fingers (score of 4), abnormal nailfold capillaries (score of 2), pulmonary arterial hypertension and/or interstitial lung disease (score of 2), and Raynaud’s phenomenon (score of 3) [2]. Our patient’s development of SSc shortly after infection with SARS-CoV-2 strongly supports the causal relationship between SSc and SARS-CoV-2 infection.

One mechanism through which SARS-CoV-2 infection is thought to lead to the development of autoimmunity is that of molecular mimicry. Molecular mimicry happens when the same lymphocyte receptor recognizes both a self-protein and a foreign antigen because of their structural similarity, which can lead to immune cross-reactivity [8]. Molecular mimicry is known to have an important role in the pathogenesis of systemic rheumatologic disorders like SLE, rheumatoid arthritis, Sjogren syndrome, and SSc [6]. One study showed that human monoclonal antibodies against SARS-CoV-2 proteins had reactivity with 28 out of 55 human antigens from a wide range of tissue groups [9]. Furthermore, another study has identified 28 human proteins with peptide sequences that are homologous to SARS-CoV-2 B cell epitopes. These proteins are known to be targeted by antibodies generated during typical autoimmune disorders, some of which have been reported to have developed after SARS-CoV-2 infection. Autoantibodies against one of these proteins, low density lipoprotein receptor 2 (LRP2), have been detected in several systemic autoimmune diseases, including SSc [10]. Such studies strongly support the theory of molecular mimicry between human and SARS-CoV-2 proteins as a mechanism leading to the development of SSc after SARS-CoV-2 infection.

Another mechanism through which SARS-CoV-2 infection is thought to result in the development of autoimmune disease is that of the formation of neutrophil extracellular traps (NETs) or NETosis [6]. NETs are networks of extracellular fibers that mainly consist of DNA and chromatin which are released from neutrophils and bind pathogens [11]. The main role of NETosis is to resolve infections by trapping microorganisms [6]. However, sustained NETosis can cause inflammation and thrombosis, and NETs can act as a source of self-antigens resulting in autoimmune disease [6,11]. Aberrant NETosis has been shown to play an important role in SSc and other autoimmune disorders, such as rheumatoid arthritis and SLE [12,13]. Excessive NETosis has been implicated in the pathogenesis of COVID-19, thus supporting the theory of NETosis as another mechanism through which SSc can develop after SARS-CoV-2 infection [14].

## Conclusions

In conclusion, our case is the second reported case of SSc developing after SARS-CoV-2 infection. As discussed above, there is strong evidence supporting molecular mimicry and NETosis as possible mechanisms through which SARS-CoV-2 infection leads to the development of SSc and other autoimmune disorders. It is likely that there will be more reports of SSc developing after SARS-CoV-2 infection.
